# Skimmed Bovine Milk-Derived Extracellular Vesicles Isolated *via* “Salting-Out”: Characterizations and Potential Functions as Nanocarriers

**DOI:** 10.3389/fnut.2021.769223

**Published:** 2021-10-28

**Authors:** Xiao-Hui Tan, Dong Fang, Yong-De Xu, Tie-Gui Nan, Wen-Peng Song, Yang-Yang Gu, Sheng-Ji Gu, Yi-Ming Yuan, Zhong-Cheng Xin, Li-Qun Zhou, Rui-Li Guan, Xue-Song Li

**Affiliations:** ^1^Department of Urology, Peking University First Hospital, Beijing, China; ^2^Institute of Urology, Peking University, Beijing, China; ^3^Beijing Key Laboratory of Urogenital Diseases (male) Molecular Diagnosis and Treatment Center, Beijing, China; ^4^Department of Urology, Beijing Friendship Hospital, Capital Medical University, Beijing, China; ^5^State Key Laboratory Breeding Base of Dao-di Herbs, National Resource Center for Chinese Materia Medica, China Academy of Chinese Medical Sciences, Beijing, China; ^6^Department of Dental Implant Center, Beijing Stomatological Hospital, School of Stomatology, Capital Medical University, Beijing, China; ^7^Department of Radiation Medicine, Institute of Systems Biomedicine, School of Basic Medical Sciences, Peking University Health Science Center, Beijing, China

**Keywords:** ammonium sulfate, extracellular vesicles, milk, proteome, RNA-seq, drug delivery systems

## Abstract

Bovine milk-derived extracellular vesicles (BM-EVs) are recognized as promising nanoscale delivery vectors owing to their large availability. However, few isolation methods can achieve high purity and yield simultaneously. Therefore, we developed a novel and cost-effective procedure to separate BM-EVs *via* “salting-out.” First, BM-EVs were isolated from skimmed milk using ammonium sulfate. The majority of BM-EVs were precipitated between 30 and 40% saturation and 34% had a relatively augmented purity. The separated BM-EVs showed a spherical shape with a diameter of 60–150 nm and expressed the marker proteins CD63, TSG101, and Hsp70. The purity and yield were comparable to the BM-EVs isolated *via* ultracentrifugation while ExoQuick failed to separate a relatively pure fraction of BM-EVs. The uptake of BM-EVs into endothelial cells was dose- and time-dependent without significant cytotoxicity. The levels of endothelial nitric oxide syntheses were regulated by BM-EVs loaded with icariside II and miRNA-155-5p, suggesting their functions as delivery vehicles. These findings have demonstrated that it is an efficient procedure to isolate BM-EVs *via* “salting-out,” holding great promise toward therapeutic applications.

## Introduction

Extracellular vesicles (EVs) are a heterogeneous group of phospholipid bilayer membrane-enclosed nanoscale particles released by various cells (1, 2). They exist in biological fluids and carry important information originating from their parent cells, which play a key and unique role in cell-cell communications. Over the past years, many biological involvements of EVs in physiological and pathological states have been established or postulated (3–5). Given the functional capabilities to transfer diverse cargo, EVs have attracted increasing interest as therapeutic agents for clinical applications such as drug delivery, immunotherapy, and cellular reprogramming (2, 6, 7). To date, several drug delivery systems have been developed and approved for clinical use, while some of them are also limited due to cytotoxicity, inefficiency, and immunogenicity (8). EVs have distinct advantages as effective vectors for delivery of drugs and functional RNAs: vast and natural sources, nano-sized dimension, protection of the cargo within a lipid bilayer, a membrane platform with ligands. However, the production of EVs from the conditioned medium is relatively cumbersome and low-yield. Food-derived EVs are considered as alternative candidates for large-scale production of EVs as they are more available and cost-effective than cell medium (9, 10). Bovine milk-derived extracellular vesicles (BM-EVs) have demonstrated biocompatibility and may serve as novel drug delivery carriers with less cytotoxic effects and immunogenic profiles (11–13). It has been thought that BM-EVs mediate immune regulation and possess anti-inflammatory properties (14). More importantly, EVs can protect the cargo from acids and enzymes in the gastrointestinal tract, meaning that oral administration is reasonably practicable (14, 15).

The ideal method to isolate EVs from other possible contaminants is required to have a high yield, purity, and scalability. Currently, commonly used techniques of EVs isolation have their own advantages and disadvantages, including ultracentrifugation and size-exclusion chromatography (SEC) (2, 16). For example, density gradient ultracentrifugation separates and concentrates EVs from other constituents based on their density, but isolates similarly sized particles with low production. Isolation kits based on the polyethylene glycol volume exclusion precipitation have been developed in recent years, yet EVs are usually contaminated with off-target protein aggregates. Salting out refers to the reduced solubility of certain molecules in a solution of high ionic strength, normally used to precipitate large biomolecules. Brownlee et al. (17) demonstrated that tumor-derived exosomes were precipitated with 0.1 M acetate and pH 4.75, and re-solubilized in acetate-free buffer at neutral pH. Ammonium sulfate is an inorganic salt with high solubility, frequently used for precipitation of the desired proteins owing to two ions that are early members of the Hofmeister series (18). Consequently, we developed a novel procedure to separate BM-EVs using ammonium sulfate. Characterizations, cellular uptake, and functional cargo delivery of the BM-EVs were investigated to indicate the feasibility of this isolation strategy.

## Materials and Methods

### Pre-treatment of Milk Samples

Skimmed bovine milk (Sanyuan Foods, Beijing, China), pasteurized and produced within 3 days, was preserved below 4°C and commercially available at a local grocery store. Defatted milk was pre-warmed for 10 min at 37°C and then mixed with 6 M hydrochloric acids (HCl) for 5 min to adjust the pH value to 4.6, followed by centrifugation at 5,000 xg for 60 min at 22°C. Casein aggregates along with other debris were discarded and the supernatant (whey, 8 mL) was added with fine-powdered ammonium sulfate to yield a 10–90% final saturation. The desired saturation to separate BM-EVs from whey proteins was determined by sodium dodecyl sulfate-polyacrylamide gel electrophoresis (SDS-PAGE).

### SDS-PAGE

Whey samples that contained 10–90% ammonium sulfate were kept at 4°C overnight and then centrifuged at 5,000 xg for 60 min at 4°C. The supernatant was collected and pellets were resuspended in phosphate-buffered saline (PBS). After adequate lysis and preparation, protein samples were loaded on a 12% polyacrylamide gel, run at 80 V, 30 min for the stacking gel and 120 V, 120 min for the separation gel, stained with coomassie brilliant blue, and visualized using an image scanner.

### Isolation of BM-EVs

#### Salting-Out

Based on the findings of SDS-PAGE, most milk proteins exist in the 10–50% supernatant solution and the 60–90% precipitation following the salting-out. Therefore, we decided to isolate BM-EVs from corresponding opposite parts. The whey samples of 10–50% ammonium sulfate and those of 60–90% were kept at 4°C for overnight and 60 min, respectively. Afterward, all whey samples were centrifuged at 5,000 xg for 60 min at 4°C. The pellets in samples of 10–50% were fully resuspended in 10 mL of 0.22-μm filtered sterile PBS and the supernatant in samples of 60–90% was simultaneously collected, prior to being filtered successively with 0.45-μm and 0.22-μm membranes. After the filtration, crude EV fractions were washed with PBS and concentrated using centrifugal ultrafiltration devices (Cat. No. MAP100C38; Pall Corp., New York, NY, USA) to obtain enriched and desalted BM-EVs.

#### Ultracentrifugation

The whey samples were filtered with 0.45-μm and 0.22-μm membranes after the removal of caseins and debris. The whey was ultracentrifuged twice at 110,000 xg for 75 min at 4°C (SW32Ti rotor, Beckman Optima L-100 XP ultracentrifuge, Brea, CA, USA). The BM-EVs pellets were resuspended in PBS and the residual precipitates were removed *via* centrifugation at 10,000 g for 5 min at 4°C.

#### Polymer-Based Precipitation

Milk samples (5 mL) were centrifuged at 3,000 xg for 15 min and filtered with 0.45-μm membranes to remove cells and debris. ExoQuick™ solution (Cat. No. EXOTC10A-1; System Biosciences, Palo Alto, CA, USA) was mixed with the supernatant and stored at 4°C overnight. The solution was then centrifuged at 1,500 xg for 30 min at room temperature and the supernatant was discarded. The pellets were further centrifuged at 1,500 xg for 5 min to remove traces of fluid and then resuspended in 600 μL of PBS.

### Characterization of BM-EVs

#### Biochemical Characterization

Protein concentrations of BM-EVs were measured using the bicinchoninic acid protein assay kit (Absin Bioscience, Shanghai, China). Analysis of EVs marker proteins was achieved *via* western blotting, using anti-CD63 (ab193349; Abcam, Cambridge, UK), anti-TSG101 (ab83, Abcam), and anti-Hsp70 (ab2787, Abcam). The presence of common co-isolated non-EV components during preparations was also probed such as Calnexin (ab75801, Abcam).

#### Physicochemical Characterization

Particle size distribution and concentrations of isolated BM-EVs were determined *via* nanoparticle tracking analysis (NTA) using NanoSight NS300 and NTA software, version 3.3 (Malvern Panalytical Ltd., Malvern, UK). The purity represented the ratio of particle number per microgram of protein (19). Transmission electron microscopy (TEM) was performed to observe the morphology of BM-EVs. EV samples were instilled onto a copper grid and then stained with 2% uranyl acetate for 5 min. Images were acquired using a digital camera mounted on a Tecnai G2 Spirit BioTwin microscope (FEI Company, Hillsboro, OR, USA) operating at 80 kV.

#### Small RNA Sequencing

The library preparation was initiated with QIAseq miRNA Library Kit (Qiagen, Hilden, Germany) following the manufacturer's instructions. Mature RNAs were ligated to specially designed adapters on their 3′ and 5′ ends and were later converted to complementary DNA with unique molecular indices using a reverse transcription primer. The complementary DNA was amplified with a universal forward primer and indexing reverse primers. After trimming adapters and filtering out low-quality reads assessed by FastQC (version 0.11.7), sequences were quantified by assigning molecules to transcript annotations and mapped to two reference databases in the following order: miRBase (miRNAs), Rfam (other small RNAs).

#### Proteomics Profiling

According to the filter-aided sample preparation protocol, the proteins of BM-EVs were precipitated with acetone, reduced, alkylated with iodoacetamide, trypsinized, and desalted. Peptide mixtures were analyzed *via* liquid chromatography-mass spectrometry (LC-MS) using Q Exactive Plus (ThermoFisher Scientific, Waltham, MA, USA) coupled to Easy-nLC 1000 (ThermoFisher Scientific). Survey full scans MS spectra at a resolution of 70,000 were acquired across the 350–1600 m/z range in a data-dependent acquisition mode. The peptide identification was performed using Proteome Discoverer software, version 2.4 (ThermoFisher Scientific).

### Cell Culture and Reagents

Human umbilical vein endothelial cells (HUVECs) were obtained from ScienCell Research Laboratories (Carlsbad, CA, USA). HUVECs were maintained in endothelial cell medium (Cat. No. 1001, ScienCell) containing fetal bovine serum (5%; Cat. No. 0025, ScienCell), endothelial cell growth supplement (1%; Cat. No. 1052, ScienCell), and antibiotic solution (1%, penicillin/streptomycin; Cat. No. 0503, ScienCell), which is a sterile and complete liquid medium designed for optimal growth of normal endothelial cells *in vitro*. Cells were incubated in a conventional humidified atmosphere at 37°C and 5% CO_2_/95% air, routinely subcultured *via* trypsinization at 90% confluency and served for later experiments within six passages.

#### Fluorescence Microscopy

For the cellular uptake *in vitro*, BM-EVs were labeled with CM-Dil (Cat. No. C7000, CellTracker^TM^; Invitrogen, Carlsbad, CA, USA) per the manufacturer's instructions. In a 96-well plate, HUVECs were contacted with CM-Dil labeled BM-EVs under indicated conditions. Before the observation by fluorescence microscopy, the cells were washed thrice with PBS and stained with 4',6-diamidino-2-phenylindole (DAPI; Beyotime Biotech, Shanghai, China) in the dark.

#### Mechanisms of Cellular Uptake

To explore the mechanisms of uptake pathway, HUVECs were pre-treated for 30 min with different inhibitors purchased from MedChemExpress (MCE, Shanghai, China) such as chlorpromazine hydrochloride (Cat. No. HY-B0407A, MCE), filipin complex (Cat. No. HY-N6716, MCE), LY294002 (Cat. No. HY-10108, MCE), dynasore (Cat. No. HY-15304, MCE). All inhibitors were dissolved in dimethyl sulfoxide (DMSO). The cells were incubated with CM-Dil labeled BM-EVs (10 μg) for 12 h and observed using a fluorescence microscope as mentioned previously.

#### Cytotoxicity Measurement of BM-EVs

To evaluate the cytotoxicity of BM-EVs, HUVECs were seeded in a 96-well plate format and treated with BM-EVs (up to 160 μg/mL) or inhibitors (chlorpromazine hydrochloride, 10 μM; filipin complex, 1 μM; LY294002, 10 μM; dynasore, 4 μM) for 12 h at 37°C and 5% CO_2_/95% air. The cell viability was measured using a Cell Counting Kit-8 (CCK-8; Keygen Biotech, Nanjing, China), with CCK-8 solution (10 μL/well) added to the cells. After a 2-h incubation at 37°C, absorbance at 450 nm was determined with a microplate reader (ThermoFisher Scientific).

### Drug Encapsulation and Functional Delivery

#### Loading Approaches

Post-loading methods such as direct co-incubation and sonication were investigated. Icariside II (Cat. No. HY-N0011, MCE) was incorporated into isolated BM-EVs. The agent dissolved in ethanol was mixed with BM-EVs suspension in the proportion of 1:9 (w/w) at room temperature, followed by co-incubation at 37°C for 2 h or sonication. Probe sonication was accomplished over ice through 30-sec bursts at 90-sec intervals with an amplitude setting of 20% (VCX 105; Sonics & Materials, Inc., Newtown, CT, USA) and repeated six times. The membrane of BM-EVs was then restored by incubating the ultrasound-treated solution at 37°C for 2 h. Most unbounded drug was removed by low-speed (10,000 xg) centrifugation for 10 min. Next, drug-loaded BM-EVs were recovered by centrifugal ultrafiltration devices (Cat. No. MCP003C46, Pall Corp.) and washed using PBS as the eluent.

#### Ultra Performance Liquid Chromatography

Loading capacity and encapsulation efficiency were calculated based on the amount of total entrapped drug per unit weight of the initially added BM-EVs and drug, respectively. Ultra-performance liquid chromatography (UPLC) was performed using the Acquity UPLC I-Class system (Waters Corp., Milford, MA, USA). The separation was performed at a flow rate of 0.4 mL/min with a gradient liquid phase of acetonitrile (A) and 0.1% formic acid (B). The gradient condition was 0–0.5 min, 25% A; 0.5–4 min, 30% A; 4–7 min, 90% A; 7–9 min 25% A. The total concentration was determined against a standard curve of reference compounds.

#### Measurement of Endothelial Nitric Oxide Synthase

The expression of endothelial nitric oxide synthase (eNOS) was determined *via* western blotting. HUVECs were treated with drug-loaded BM-EVs for 2 days, which were later suspended in lysis buffer and incubated on ice for 30 min. Protein samples were separated using 10% polyacrylamide gel, transferred to a polyvinylidene fluoride membrane, blocked with 5% (w/v) skimmed milk for 1 h at room temperature, and blotted overnight at 4°C with primary antibodies against eNOS (Cat. No. 5880; Cell Signaling Technology, Danvers, MA, USA) and β-actin (ab8226, Abcam). After incubation with secondary antibodies and acquiring images, the band intensity was quantified using ImageJ software (NIH, Bethesda, MD, USA).

#### Determination of Intracellular Nitric Oxide

HUVECs were seeded in a 96-well plate and treated with PBS, BM-EVs, free drug, and drug-loaded BM-EVs for 24 h. Intracellular nitric oxide (NO) content was detected by the probe 3-Amino,4-aminomethyl-2',7'-difluorescein, diacetate (DAF-FM DA; Beyotime Biotech) according to the manufacturer's protocol.

### Loading of Nucleic Acid Cargo Into BM-EVs

#### Chemical Transfection

Exogenous siRNA/miRNA was incorporated into BM-EVs using the Exo-Fect^TM^ (Cat. No. EXFT200A-1; System Biosciences). The Exo-Fect was initially added with Cy3-labeled transfection control or siRNA/miRNA and then incubated with BM-EVs (150–300 μg in 100 μL) in the dark.

#### Packaging siRNA Into BM-EVs

For the knockdown of gene expression of glyceraldehyde 3-phosphate dehydrogenase (GAPDH), HUVECs were seeded in 6-well plates the day before and treated with PBS or BM-EVs samples for 48 h. The sense sequences of chemically synthesized siRNA oligos (GenePharma, Shanghai, China) were as follows: scrambled siRNA, 5′-UUC UCC GAA CGU GUC ACG UTT-3′; GAPDH siRNA, 5′-UGA CCU CAA CUA CAU GGU UTT-3′. The knockdown effect was evaluated *via* western blotting, using anti-GAPDH (ab181602, Abcam) and anti-β-actin (ab8226, Abcam).

#### Functional Delivery of miRNAs *via* BM-EVs

To assess the regulation of eNOS by loaded miRNAs, HUVECs were seeded into 6-well plates and maintained in endothelial cell medium for 1 day. The cells were treated using BM-EVs transfected with agomiR-155-5p (duplex oligos sense, 5′-UUA AUG CUA AUC GUG AUA GGG GUU-3′, 5′-CCC CUA UCA CGA UUA GCA UUA AUU-3′), NC agomir (duplex oligos sense, 5′-UUC UCC GAA CGU GUC ACG UTT-3′, 5′-ACG UGA CAC GUU CGG AGA ATT-3′), antagomiR-155-5p (sense, 5′-AAC CCC UAU CAC GAU UAG CAU UAA-3′), and NC antagomir (sense, 5′-CAG UAC UUU UGU GUA GUA CAA-3′). The measurements of eNOS were performed using western blotting as described previously.

### Statistical Analysis

All quantitative variables were presented as mean ± standard deviation. Differences between two groups were determined using unpaired Student's *t*-test. Analysis of variance (ANOVA) was performed for an overall difference of multiple groups and, if significant, followed by Tukey's multiple comparisons between groups. The enriched pathways and potential functions of identified differentially expressed genes were analyzed using Kyoto Encyclopedia of Genes Genomes (KEGG) as well as Gene Ontology (GO). The significance level of enrichment was assessed by Fisher's exact test. Statistical analyses were performed using SAS^®^ software, version 9.4 (SAS Institute, Cary, NC, USA). *P*-values <0.05 were considered statistically significant.

## Results

### Isolation of BM-EVs

**Figure 1:**
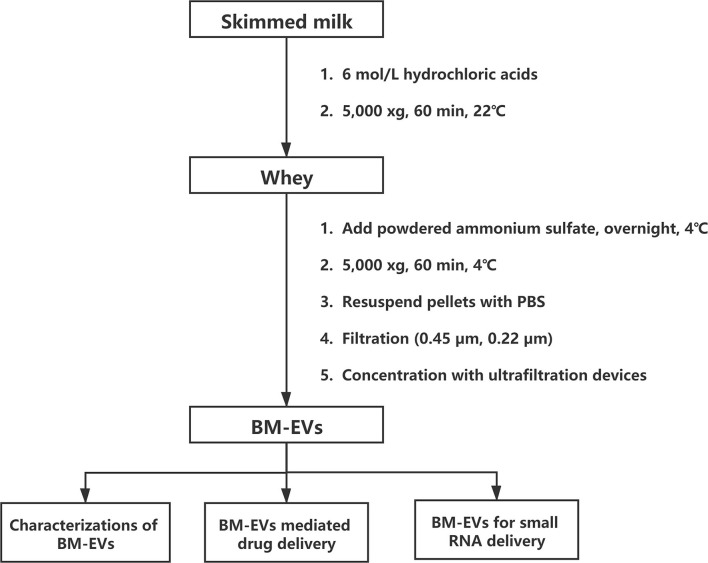
Flowchart of isolating BM-EVs with ammonium sulfate.

**Figure 2:**
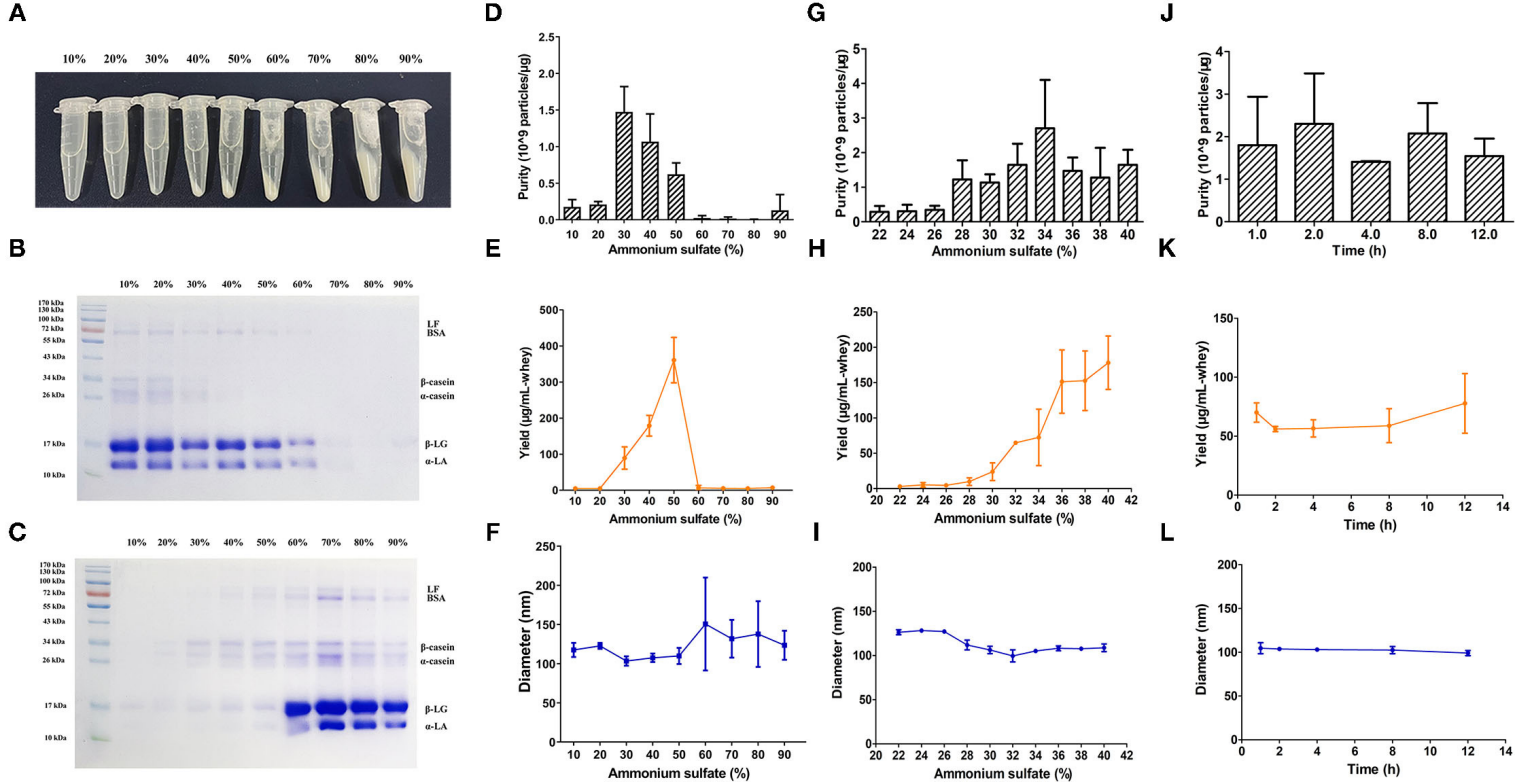
Isolation of BM-EVs *via* salting-out. **(A)** The precipitation of whey proteins was achieved with different saturation of ammonium sulfate. The SDS-PAGE of milk proteins in the supernatant **(B)** and the pellets **(C)** following the salting-out were visualized using an image scanner. BM-EVs separated from different saturation and duration were characterized by purity **(D,G,J)**, yield **(E,H,K)**, diameter **(F,I,L)**. Data were presented as mean ± standard deviation (*n* = 3). LF, lactoferrin; BSA, bovine serum albumin; β-LG, β-lactoglobulin; α-LA, α-lactalbumin.

The isolation of BM-EVs from purchased bovine milk was implemented with a combination of acidification and salting-out effects (Figure 1). The protein and fat content of milk samples was 3.1% and below 0.3%, respectively. The precipitation of casein was performed with HCl to obtain the supernatant whey. Different amounts of ammonium sulfate were mixed with whey, which resulted in more pellets as the salt concentrations increased (Figure 2A). The SDS-PAGE analysis revealed that major non-EVs proteins of bovine milk were soluble in 10–50% ammonium sulfate and precipitated in the 60–90% saturation (Figures 2B,C). The purity of BM-EVs reached a peak in the pellets of 30% ammonium sulfate with a moderate yield, in contrast to an insignificant production in the supernatant of 60–90% ammonium sulfate (Figures 2D,E). The mean diameter of BM-EVs isolated from 10 to 50% saturation fluctuated between 100 nm and 150 nm (Figures 2F,I). In light of these findings, the pellets from 20–40% ammonium sulfate were further investigated to optimize the condition. The purity of BM-EVs isolated using 34% saturation was higher than the others and the yield began to grow from 26% (Figures 2G,H). As for the duration of salting out, BM-EVs were generally precipitated after 1 h, and the yield generally ranged from 50 and 100 μg/mL-whey within 12 h (Figures 2J–L). Therefore, 34% ammonium sulfate and 12-h salting-out were adopted in the following studies.

### Characterizations of BM-EVs

**Figure 3:**
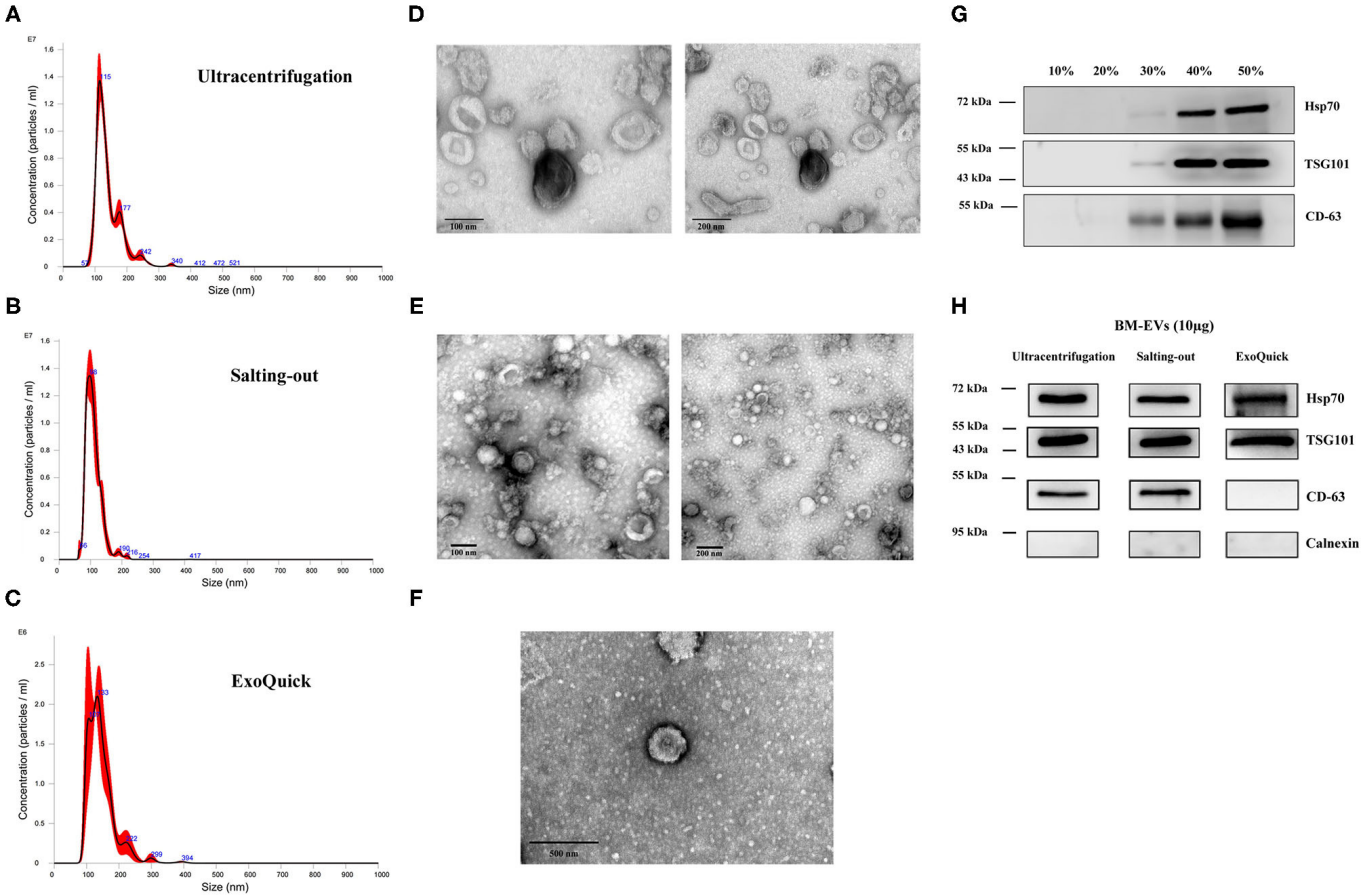
Characterization of BM-EVs. The size distribution of isolated BM-EVs was analyzed by nanoparticle tracking analysis **(A–C)**. Transmission electron microscopy observation of BM-EVs separated by three different methods **(D–F)**. The protein markers (CD63, TSG101, Hsp70) of BM-EVs using different saturation of ammonium sulfate **(G)**. Western blotting of BM-EVs isolated *via* salting-out, ultracentrifugation or ExoQuick method **(H)**.

Three milk samples of different expiration dates were used as biological replicates, and the mean particle/protein ratio was 1.68 ± 0.17 × 10^9^ particles/μg-protein with a yield of 61.105 ± 22.016 μg/mL-input skimmed milk (Table 1). The purity and yield of salting-out method were comparable to the BM-EVs isolated *via* ultracentrifugation (*P* > 0.05 for both), whereas ExoQuick alone failed to separate a relatively pure fraction of BM-EVs owing to protein contamination (*P* = 0.0009). The size distribution measured by NTA indicated that a homogeneous population of BM-EVs *via* different methods was largely 70–200 nm in diameter (Figures 3A–C). The surface morphology of BM-EVs separated *via* ultracentrifugation and salting-out showed a spherical shape with a diameter of ~100 nm while those isolated by ExoQuick did not have a “cup-shaped” appearance through the observation of TEM (Figures 3D–F). The BM-EVs separated from 30–50% saturation contained EVs marker proteins such as CD63, TSG101, and Hsp70, which also increased with the percentage saturation of ammonium sulfate (Figure 3G). The marker proteins of BM-EVs were verified with western blotting for CD63, TSG101, and Hsp70 (Figure 3H). Interestingly, CD63 was not enriched in BM-EVs isolated *via* ExoQuick. The endoplasmic reticulum marker Calnexin, as expected, was absent from the BM-EVs fraction (Figure 3H).

**Table 1:** Separation of BM-EVs *via* different isolation techniques.

	**Salting-out**	**Ultracentrifugation**	**ExoQuick**	***P*** **values**
Purity (10^9^ particles/μg)	1.677 ± 0.173	2.116 ± 0.561	0.124 ± 0.034	0.3219
				0.0033
				0.0009
Yield (μg/mL-input skimmed milk)	61.105 ± 22.016	51.909 ± 3.525	1,283.37 ± 6.635	0.6949
				<0.0001
				<0.0001
Particle yield (10^11^ particles/mL-input skimmed milk)	1.046 ± 0.466	1.086 ± 0.22	1.594 ± 0.442	0.9918
				0.2767
				0.3205
Diameter (nm)	97.833 ± 3.609	129.433 ± 4.179	139.033 ± 4.751	0.5391
				0.0219
				0.5391

*Data are presented as mean ± standard deviation (n = 3)*.

*P-values are listed in the following order from the top: salting-out vs. ultracentrifugation, salting-out vs. ExoQuick, and ultracentrifugation vs. ExoQuick*.

**Figure 4:**
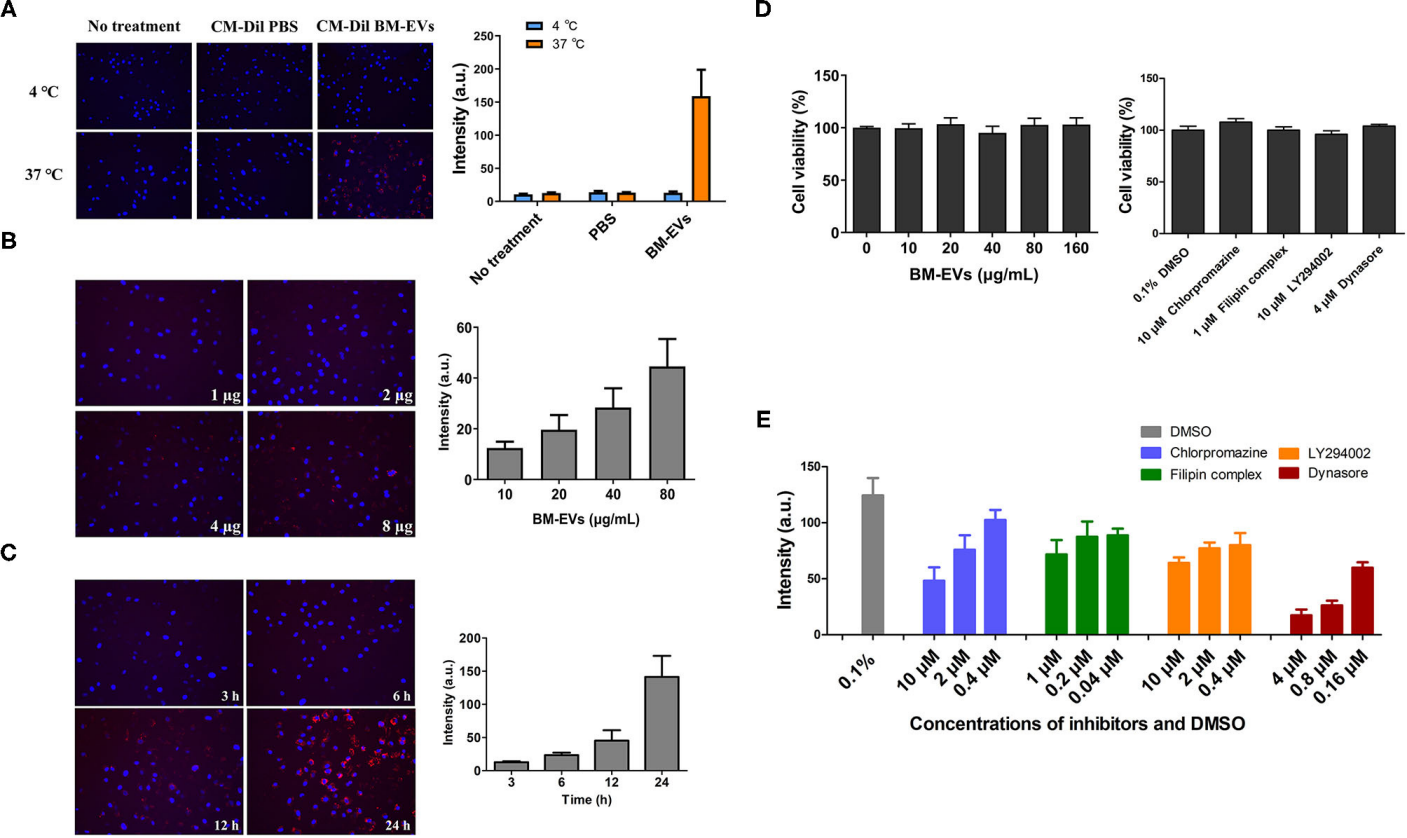
Cellular uptake and cytotoxicity of BM-EVs. **(A)** HUVECs were incubated with 20 μg CM-Dil labeled BM-EVs (red) at 37 or 4°C and monitored after 3 h. The cells were untreated or treated with CM-Dil PBS as a negative control. Nuclei were visualized by staining with DAPI (blue). Representative images are shown (left panel) and fluorescent intensity of CM-Dil labeled BM-EVs was quantified using Image-Pro Plus software (right panel; n = 3, mean ± standard deviation). **(B)** CM-Dil labeled BM-EVs at 0-80 μg/mL were added to HUVECs in a 96-well plate and monitored after 12 h (*n* = 3, mean ± standard deviation). **(C)** CM-Dil labeled BM-EVs were added at 80 μg/mL and monitored after 3, 6, 12, and 24 h (*n* = 3, mean ± standard deviation). **(D)** Cytotoxicity of BM-EVs (left panel) and inhibitors (right panel) *in vitro*: HUVECs were treated with BM-EVs or inhibitors for 12 h in a conventional incubator (*n* = 4, mean ± standard deviation). **(E)** The uptake of CM-Dil labeled BM-EVs (10 μg) was assessed after 12 h in the presence of 0.1% DMSO, chlorpromazine (hydrochloride), filipin complex, LY294002, and dynasore (*n* = 3, mean ± standard deviation). All images were observed using 200× fluorescence microscopy.

The cellular uptake of BM-EVs was investigated *in vitro*, in which the concentrated fraction for BM-EVs was fluorescently labeled with the membrane dye CM-Dil. HUVECs were treated with CM-Dil labeled BM-EVs at various temperatures, doses, and duration. After co-incubation for 3 h at 37°C, an abundant signal of fluorescence was observed inside the cells while the negative controls showed no significant uptake (Figure 4A). Moreover, the cellular internalization was substantially inhibited at 4°C in contrast with 37°C, suggesting that an active and energy-dependent process was involved. Dose-dependent and time-dependent increases in BM-EVs uptake were observed until 80 μg/mL and 24 h (Figures 4B,C). The cytotoxic effect of BM-EVs or inhibitors (chlorpromazine hydrochloride, filipin complex, LY294002, dynasore) on HUVECs was compared with controls and the cell viability did not change significantly (*P* > 0.05 for both), nearly remaining at 100% (Figure 4D). To unravel the specific pathways behind it, several inhibitors were used to block endocytosis and phagocytosis, showing that the fluorescence decreased vs. 0.1% DMSO and depended on their concentrations (Figure 4E).

**Figure 5:**
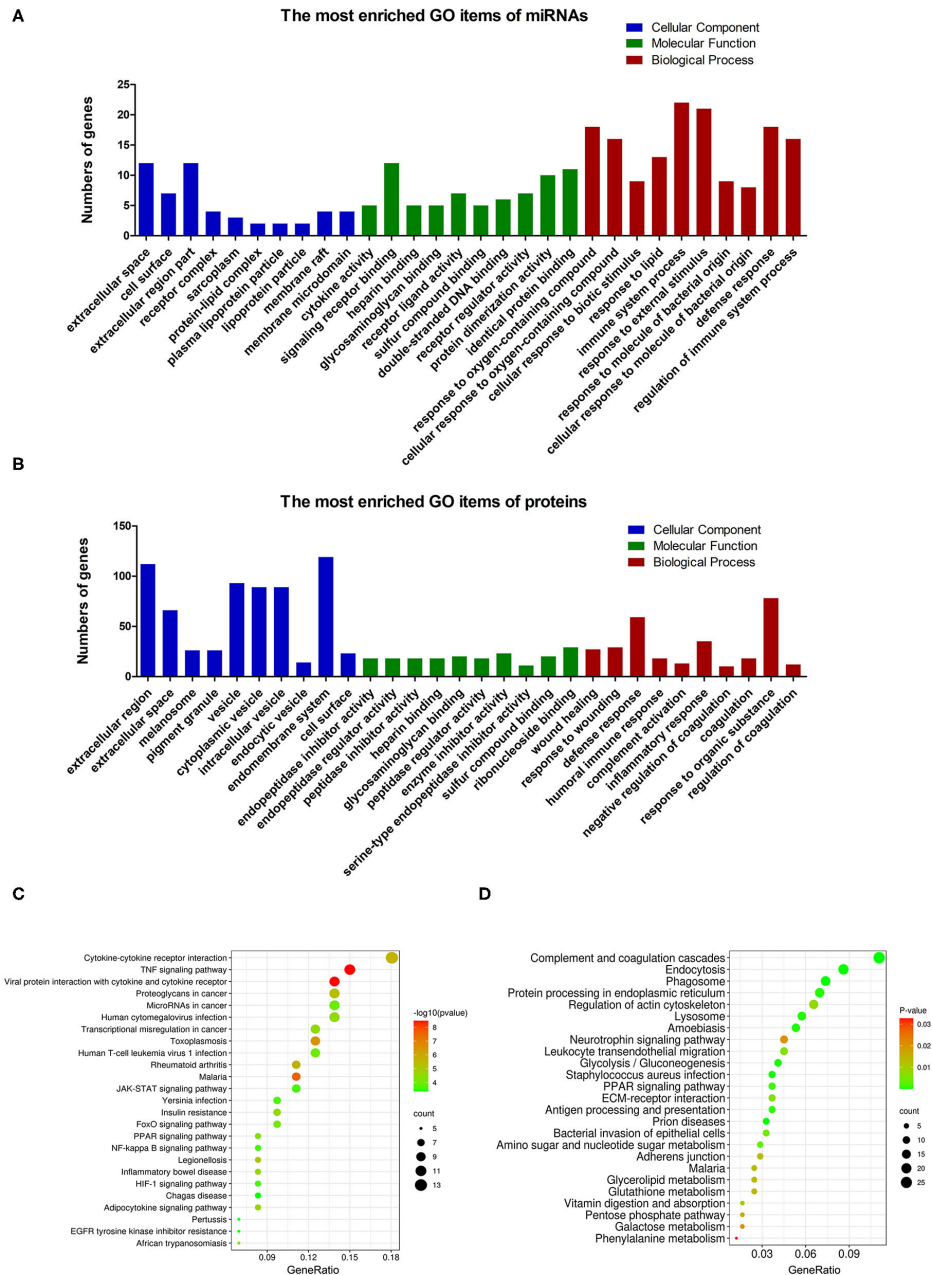
Bioinformatic analysis based on small RNA sequencing and proteomic profiling of BM-EVs. GO enrichment analysis of the differentially regulated microRNAs **(A)** and proteins **(B)** was performed. The enriched terms were ranked in ascending order of P-values from left to right, indicating that the P-value of the first column in each section was the minimum. The enriched KEGG pathways of the differentially regulated microRNAs **(C)** and proteins **(D)** were illustrated using a bubble plot.

The proteome analysis of BM-EVs from three samples using LC-MS detected 579 proteins in total, some of which were EVs-associated markers and milk proteins (Table 2; Supplementary Table 1). These findings indicated that the membrane proteins of BM-EVs could be detected following acidification and salting-out processes. Next-generation RNA sequencing was performed to catalog small RNAs from BM-EVs, showing an enrichment of small RNA molecules <200 nucleotides. Thousands of miRNAs were identified and the most abundantly expressed were let-7b, let-7a-5p, miR-148a, miR-3596, and miR-200c (Supplementary Table 2). The percentage distribution of small RNA biotype counts was as follows: miRNA (12.97%), tRNA (62.93%), snoRNA (5.81%), rRNA (5.12%), snRNA (7.22%), and others (5.45%) (Supplementary Table 3). GO enrichment analysis revealed the potential functions of target miRNAs and proteins (Figures 5A,B). Notably, the terms related to EVs in the cellular component were significantly enriched, including extracellular region, vesicle, and endomembrane system. Meanwhile, miRNAs differentially enriched in BM-EVs were associated with biomolecule binding, immune system, and response to the stimulus. According to KEGG pathway analysis, target genes were mainly involved in cellular interaction and inflammation (Figures 5C,D).

**Table 2:** Summary of BM-EVs proteome profiling.

**Group**	**Family**	**Proteins**
EVs-associated proteins	Tetraspanins	CD9, CD63, CD81
	Multi-pass membrane proteins	CD47
	Heat shock proteins	HSP70 (HSPA1A, HSPA13, HSPA2), HSP90 (HSP90AA1, HSP90AB1), HSC70 (HSPA8)
	GPI-anchored 5' nucleotidase	NT5E
	Rab proteins	RAB18, RAB1B, RAB7A, RAB11B, RAB5C, etc.
	Annexins	Annexin A5, A1, A2, A4, A7, A11, A3
	Others	ADAM10, EPCAM, CHMP3, CHMP1B, FLOT1, RHOA, etc.
Milk proteins	Caseins	αS1, αS2, β, and κ casein
	Albumins	bovine serum albumin, α-lactalbumin
	Others	β-lactoglobulin, TGF-β, etc.
Others	lipoproteins	lipoprotein A-I, A-II, A-IV, C-II, C-III, C-IV, D, E

### BM-EVs as Drug Delivery Vesicles

**Figure 6:**
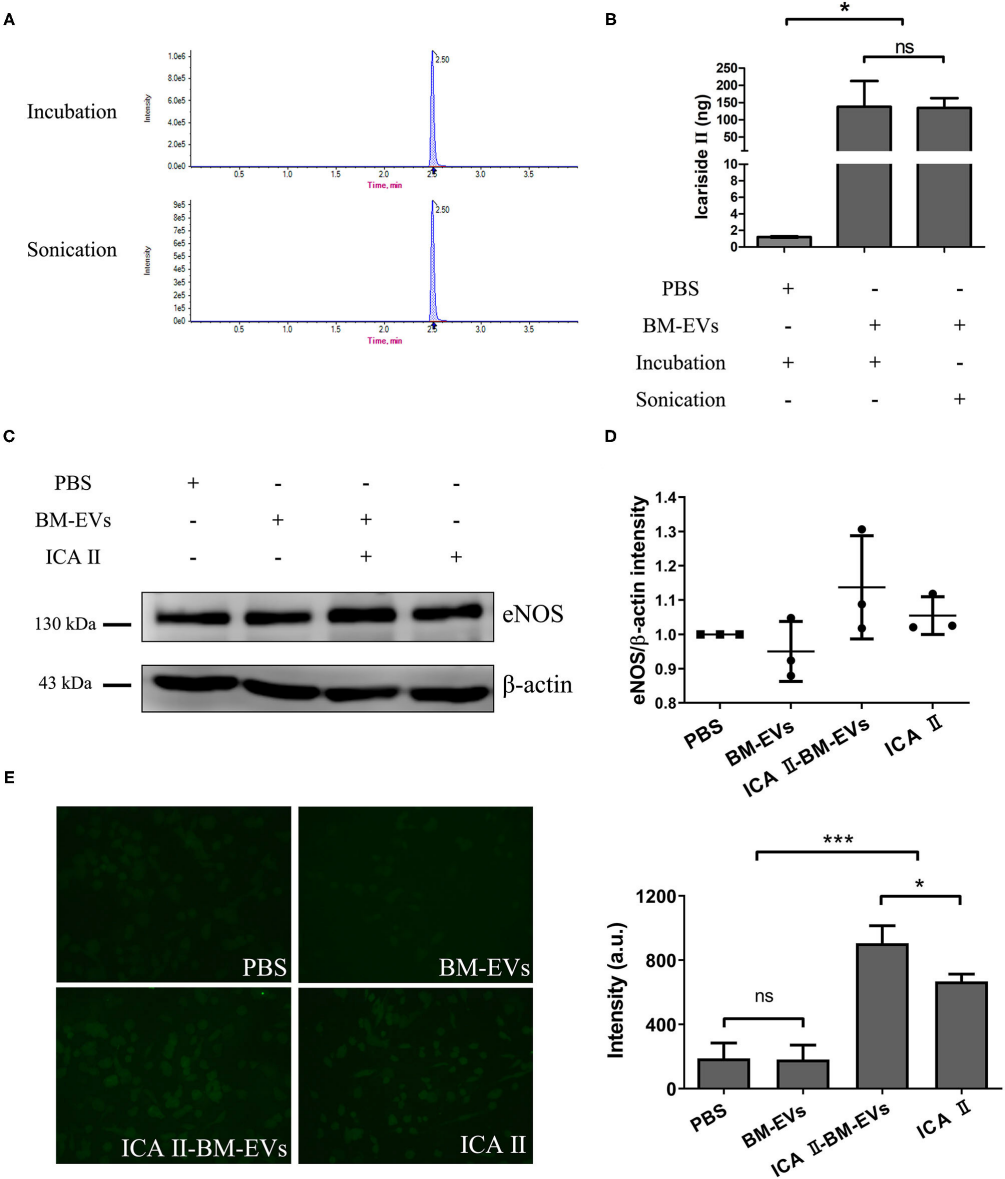
Drug encapsulation and functional delivery mediated by BM-EVs. **(A)** UPLC profile of ICA II loaded into BM-EVs *via* incubation or sonication. **(B)** After the initial mixture with 10 μg drug, the content of ICA II was determined *via* UPLC following the separation from free ICA II. **(C)** After 48-h incubation with ICA II-loaded BM-EVs or controls as indicated, the proteins of eNOS and β-actin in HUVECs were evaluated by western blotting. **(D)** The density in contrast to the PBS control was quantified using ImageJ software (*n* = 3, mean ± standard deviation). **(E)** Intracellular NO levels were determined using the probe DAF-FM DA in HUVECs treated with PBS, BM-EVs, ICA II, and ICA II-loaded BM-EVs. Representative images observed using a 200× fluorescence microscopy are shown (left panel) and fluorescent intensity was quantified (right panel; n = 3, mean ± standard deviation). ICA II, icariside II; ns, *P* > 0.05; *, *P* ≤ 0.05; ***, *P* ≤ 0.001.

The BM-EVs-bound icariside II was formulated *via* incubation and sonication, both of which showed the same retention time in UPLC profiles (Figure 6A). According to the measurements, BM-EVs have significantly increased the amount of icariside II as compared with PBS, indicating that removal of free drug and formulation of drug-loaded BM-EVs were both accomplished (Figure 6B). Loading capacity and encapsulation efficiency of BM-EVs loaded with icariside II by incubation were 1.00 ± 0.58% and 1.38 ± 0.75%, respectively. Similarly, sonication led to the loading capacity of 0.93 ± 0.34% and the encapsulation efficiency of 1.34 ± 0.49%. The treatment of icariside II alone improved the expression of eNOS in the HUVECs at a concentration of 0.5 μM. Drug-loaded BM-EVs that encapsulated equivalent icariside II exhibited enhanced effects on up-regulating the levels of eNOS (*P* = 0.1529, Figures 6C,D). Measurements of intracellular NO revealed that the endothelial NO levels were fairly elevated using icariside II compared with PBS or BM-EVs (*P* = 0.0007). In addition, BM-EVs formulation showed increased efficacy as compared with icariside II alone (*P* = 0.0433, Figure 6E).

**Figure 7:**
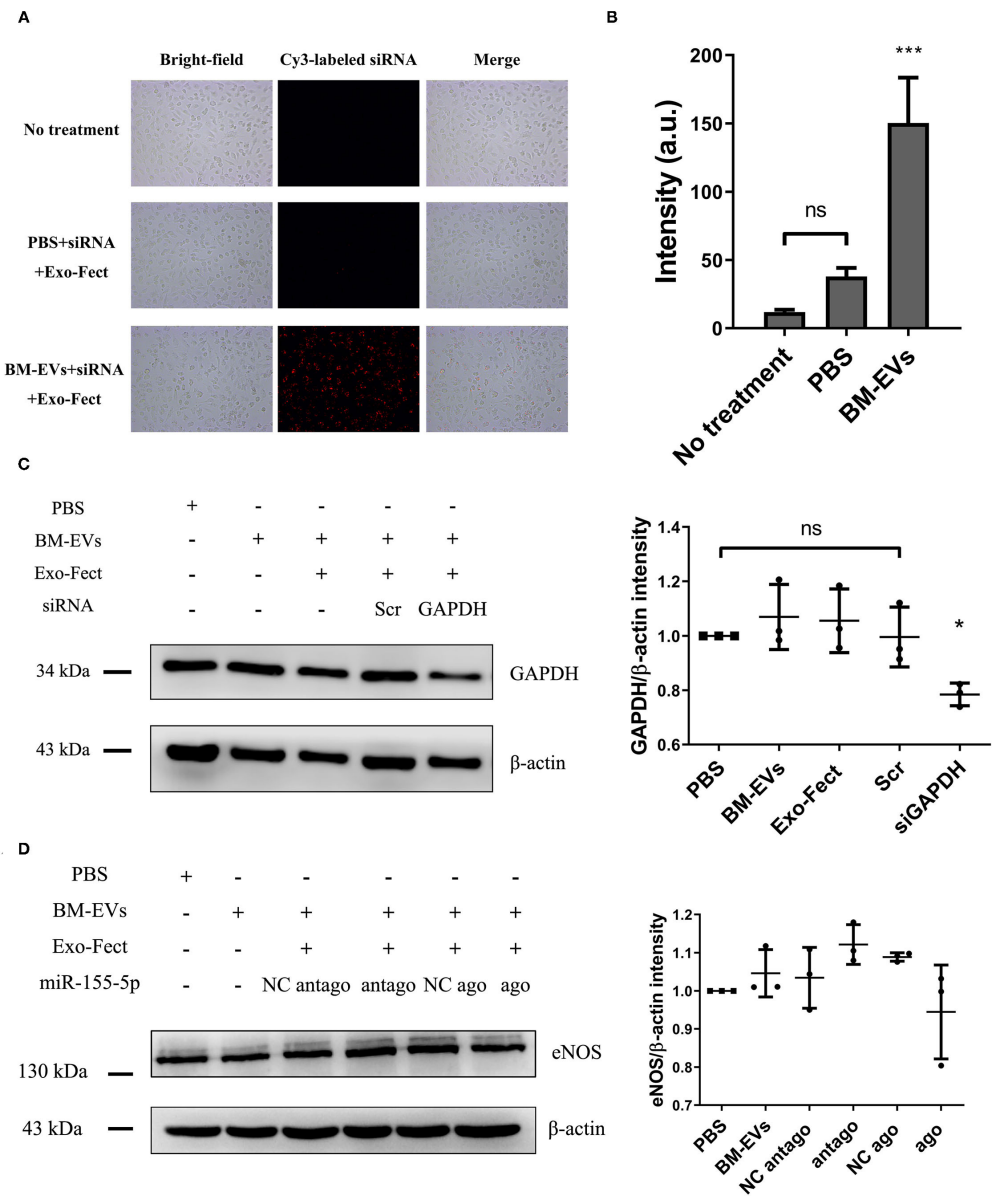
Loading of nucleic acid cargo into BM-EVs by Exo-Fect. **(A)** siRNA (Cy3-labeled transfection control) was loaded in BM-EVs (150–300 μg in 100 μL) by Exo-Fect, delivered to HUVECs, and visualized after 12 h transfection. Shown are the bright-field images (left panels), fluorescence images (middle panels), and merged images (right panels). **(B)** Fluorescent intensity of Cy3-labeled siRNA was quantified using Image-Pro Plus software. **(C)** After 48-h incubation with modified BM-EVs or controls as indicated, the proteins of β-actin and GAPDH in HUVECs were evaluated by western blotting (*n* = 3, mean ± standard deviation). **(D)** Levels of eNOS and β-actin were measured in HUVECs treated with BM-EVs containing agomiR-155-5p, antagomiR-155-5p and controls (*n* = 3, mean ± standard deviation). Scr, scrambled siRNA; NC, negative control; ago, agomiR-155-5p; antago, antagomiR-155-5p; ns, *P* > 0.05; *, *P* ≤ 0.05; ***, *P* ≤ 0.001.

Exogenous loading of small RNA cargo into BM-EVs was achieved by the chemical transfection agent, Exo-Fect. Compared with controls, BM-EVs treatment resulted in an increased signal of fluorescence, indicating a successful transfer of Cy3-labeled siRNA to HUVECs (*P* = 0.0003, Figures 7A,B). Delivery of siRNA targeted to GAPDH in the cells silenced the genes by nearly 30% vs. controls (*P* = 0.0213). On the other hand, PBS, BM-EVs, Exo-Fect, and scrambled siRNA had no significant effect on the GAPDH expression (Figure 7C). After 48-h treatment of BM-EVs loaded with agomiR-155-5p, the eNOS protein expression in HUVECs was reduced compared with negative controls, whereas those contained antagomiR-155-5p promoted the levels of eNOS (*P* = 0.0875, Figure 7D). Taken together, these data supported that BM-EVs isolated by ammonium sulfate hold therapeutic implications as potential vehicles for the delivery of therapeutically active molecules, including synthetic drugs, siRNAs, and miRNAs.

## Discussion

We have described the development of a salting-out method to isolate BM-EVs from whey and provided preliminary evidence of their function as therapeutic carriers. The BM-EVs precipitated by ammonium sulfate have several characterizations that were consistent with previous knowledge, including nanoscale spherical shape, proteins, and nucleic acid cargo. The effects of acidification on BM-EVs and their resistance against digestion have been studied previously, suggesting that BM-EVs hold a stable property under harsh degrading conditions (20–22). In our study, BM-EVs in the solution of HCl and ammonium sulfate could be recovered using centrifugal ultrafiltration devices, which showed their ability to withstand high ionic strength as both chemicals are adequately soluble in water. The salting-out method had regular characterizations shared with ultracentrifugation, including protein markers, morphology, etc. Although ExoQuick has been a widely used kit in the field of EVs, it is not sufficient for BM-EVs separation in bovine milk owing to the precipitation of many contaminating proteins, especially caseins. We found that the BM-EVs isolated with 34% saturation have a relatively robust purity and yield as compared with ultracentrifugation. The specific ratios were both ~2 × 10^9^ particles/μg-protein and 70 μg/mL-whey. Moreover, the yield of BM-EVs has been obviously increased until 40% saturation, reaching 200 μg/mL-whey with a decent purity of 1 × 10^9^ particles/μg-protein. In our opinion, the majority of BM-EVs was precipitated between 30% and 40% saturation. The duration of salting-out process was also investigated in another independent experiment, ranging from 60 min to overnight. Normally, the saturated solution appears to be fully equilibrated in 1 h. The duration was prolonged in our study to investigate whether BM-EVs can remain active and functional for 12 h in the acidic and saline solution. This protocol was rather cost-effective and did not require specialized equipment. The major expenses were covered for ultrafiltration centrifugal filters instead of ultracentrifugation devices, SEC columns, or commercially available kits. Differential centrifugation followed by density gradient ultracentrifugation has been another efficient procedure to isolate milk EVs (23). Compared with ultracentrifugation, an additional density gradient step can further increase the purity. More research is needed to testify whether this separation strategy can be helpful to optimize the salting-out method.

We observed the uptake and biocompatibility of BM-EVs in HUVECs. HUVECs are primary cells isolated from the vein of the umbilical cord, serving as a model system for studies of the function and pathology of endothelial cells, such as cardiovascular disorders, tumor-associated angiogenesis, oxidative stress, and erectile dysfunction. First, low temperature apparently reduced the uptake of BM-EVs, showing that energy-dependent endocytosis pathways were likely to be involved in the internalization of BM-EVs. Second, a dose- and time-dependent uptake of BM-EVs was observed with no cytotoxicity, even at a higher concentration. Similar patterns of BM-EVs uptake have been reported in Raw264.7 cells and cancer cells (11, 13). The main routes and mechanisms of EVs uptake have been well studied and characterized, including membrane fusion, phagocytosis, macropinocytosis, endocytosis (24). In particular, endocytosis consists of clathrin-mediated endocytosis, caveolin-dependent endocytosis, and lipid raft-mediated endocytosis. Many molecular inhibitors appear to reduce EVs uptake, normally used to demonstrate the routes through which recipient cells internalize EVs. In our study, the uptake of BM-EVs was partially inhibited by different concentrations of chlorpromazine, dynasore, LY294002, filipin, which blocked endocytosis and phagocytosis. These findings support that the uptake and underlying mechanisms of BM-EVs separated by ammonium sulfate were in accordance with previous research.

The proteome profiling and small RNA sequencing have further shed light on the integral constituents of BM-EVs isolated by the salting-out method. The proteomic analysis has revealed that the BM-EVs contain tetraspanins, annexins, heat shock proteins, and other EV markers. The presence of milk proteins such as casein, lactalbumin, lactoglobulin, lactoferrin, and TGF-β has also been detected. Several proteomic studies for BM-EVs have been performed and there is hardly a discrepancy as compared with our findings (11, 15). Meanwhile, profiling of small RNAs and miRNAs in BM-EVs was also similar to previous reports (25, 26). GO term and KEGG pathway enrichment analysis showed that differentially expressed miRNAs and proteins in BM-EVs were related to response to the stimulus, cancer, immunity, and metabolism-associated pathways, which was consistent with current evidence of many pathophysiological processes regulated by BM-EVs (27).

Icariin is a principal bioactive compound extracted from traditional Chinese medicine *Epimedium brevicornum*, having a variety of pharmacological effects that could be used for the treatment of erectile dysfunction, neurodegenerative disease, and cardiovascular disorders (28, 29). One of its metabolites, icariside II, has been found to inhibit phosphodiesterase-5, regulate the expression of eNOS, and enhance the production of NO (30, 31). However, several obstacles restrict its further clinical translation, including poor aqueous solubility, low membrane permeability, and obvious efflux from cells. Synthetic micelles for encapsulation and delivery of icariside II have provided a rationale to address these issues (32, 33). EVs have promising potentials as nano-sized drug delivery systems due to their natural origin and high biocompatibility, which can increase drug efficacy and minimize off-target side effects. It has been analyzed that drug release from BM-EVs could be controlled over a period of 24–48 h, implying that drugs can efficiently reach the target with longer circulation time and higher stability (13). The incorporation of drug molecules into EVs may be influenced by loading methods, drug properties, and EVs/drug proportion (34). It has been reported that sonication has been a more efficient procedure as compared with incubation and electroporation (35). Although icariside II was successfully loaded into BM-EVs in our study, loading capacity and encapsulation efficiency were both merely about 1% *via* incubation or sonication. In our analysis, the fluidity of membranes was not investigated because a longer duration of restoration was used compared with Kim et al. (35). They demonstrated significant decreases in membrane microviscosity upon sonication and a complete restoration of membrane microviscosity after a 1-h incubation at 37°C. To date, many studies applied BM-EVs as a novel drug delivery system, using curcumin, paclitaxel, docetaxel, celastrol, etc. Different loading methods and parameters have a considerable effect on drug incorporation into EVs, resulting in variable outcomes (34). We argue that the properties of icariside II and specific procedure details may impact the efficiency of encapsulation. Though the up-regulation of eNOS by icariside II and drug-loaded BM-EVs was not of statistical significance, endothelial NO was significantly elevated. The inconsistent results may be attributed to the semi-quantitative data measured by western blot and fluorescence images. Further optimization of encapsulation methods and application of other bioactive agents are required to explore the role of BM-EVs in drug delivery systems.

At present, increasing attention has been focused on the applications of EVs as vehicles to deliver RNA therapeutics such as siRNAs, miRNAs, and mRNAs (36). For instance, EVs derived from normal fibroblast-like mesenchymal cells, electroporated with siRNA targeting oncogenic mutant KrasG12D, demonstrated cancer growth suppression and increased survival in several mouse models of pancreatic cancer (37). It has also been shown that BM-EVs are a favorable nano-carrier for the siRNA delivery against multiple cancer cells (12). As an essential regulator of eNOS and NO, miR-155 may play an important role in endothelial dysfunction, and inhibition of its expression could be a new therapeutic approach (38). In our study, the protein expression of eNOS and GAPDH was possibly regulated by BM-EVs transfected with miR-155-5p and siRNA targeting GAPDH, respectively. The difference of statistical significance probably resulted from the subjective quantification of western blot. Successful loading of siRNA or miRNA into BM-EVs was achieved using Exo-Fect, which is another strong evidence that BM-EVs precipitated by ammonium sulfate have functional potential to transfer therapeutic entities. Moreover, BM-EVs can be further engineered to obtain a desired trait. Del Pozo-Acebo et al. reported that BM-EVs could be used as potential nanocarriers for RNA-based therapy with a biological effect through the modulation of gene expression (39). It is acknowledged that bioengineered EVs can express specific surface molecules and bind therapeutic RNA, making them an encouraging membrane platform in the future (6).

Several principle limitations of our study are noteworthy. First, the release profile of BM-EVs loaded with icariside II was not determined. Nonetheless, the drug release studies may not necessarily reflect the actual drug release from EVs since drug molecules are likely to adhere to the surface of EVs (40). Second, lack of direct comparisons with other loading techniques would make it difficult to optimize current protocols. Third, ammonium sulfate may impact the components within EVs, restricting the scope of clinical application to a certain extent. Moreover, the functions of engineered BM-EVs warrant more evidence to maximize the potential efficacy. The ideal separation method needs to achieve high throughput while ensuring high purity and low damage. Obviously, current strategies are unlikely to reach this goal. From our perspective, ultracentrifugation, also known as differential centrifugation, is still preferred at present, and the salting-out method can provide an additional option in EVs research. Further efforts are required to address these problems, offering valuable insights into this potent candidate for translation from the bench to the bedside.

In summary, we developed a novel procedure to isolate BM-EVs using the salting-out effect of ammonium sulfate and validated their function as therapeutic delivery vehicles. Although existing research has obtained inspiring findings, our understanding of the underlying mechanisms still remains in its infancy. Hopefully, collaboration among cell biologists, physicians, and biopharmaceutical companies in a complementary manner will render BM-EVs another weapon to the armory for combating diseases.

## Data Availability Statement

Data are available on reasonable request. All data generated or analyzed during this study are included in this published article and its supplementary information files. The raw mass spectrometry proteomics data have been deposited to the ProteomeXchange Consortium *via* the PRIDE partner repository with the dataset identifier PXD028095. RNA-Seq data have been available in the Gene Expression Omnibus (GEO) database (http://www.ncbi.nlm.nih.gov/geo/) under the accession number GSE183175.

## Ethics Statement

Ethical approval was waived since this article does not contain any studies with human participants or animals.

## Author Contributions

R-LG and X-SL: conception and design. Z-CX, L-QZ, and X-SL: administrative support. X-HT, T-GN, W-PS, Y-YG, S-JG, and Y-MY: collection and assembly of data. X-HT, DF, and Y-DX: data analysis, interpretation, and manuscript writing. All authors contributed to manuscript revision, read, and approved the submitted version.

## Funding

Supported by the National Natural Science Foundation of China (Grant No. 81971379) and Beijing Natural Science Foundation (Grant No. L182004).

## Conflict of Interest

R-LG has patent pending to China National Intellectual Property Administration. The remaining authors declare that the research was conducted in the absence of any commercial or financial relationships that could be construed as a potential conflict of interest.

## Publisher's Note

All claims expressed in this article are solely those of the authors and do not necessarily represent those of their affiliated organizations, or those of the publisher, the editors and the reviewers. Any product that may be evaluated in this article, or claim that may be made by its manufacturer, is not guaranteed or endorsed by the publisher.

## Abbreviations

ANOVA: analysis of variance
CCK-8: cell counting kit-8
DAF-FM DA, 3-amino, 4-aminomethyl-2', 7'-difluorescein: diacetate
DAPI, 4': 6-diamidino-2-phenylindole
DMSO: dimethyl sulfoxide
eNOS: endothelial nitric oxide synthase
EVs: extracellular vesicles
BM-EVs: bovine milk-derived extracellular vesicles
GAPDH: glyceraldehyde 3-phosphate dehydrogenase
GO: gene ontology
HCl: hydrochloric acids
HUVECs: human umbilical vein endothelial cells
KEGG: Kyoto encyclopedia of genes genomes
LC: liquid chromatography
MS: mass spectrometry
miRNA: microRNA
NO: nitric oxide
NTA: nanoparticle tracking analysis
PBS: phosphate-buffered saline
siRNA: small interfering RNA
SDS-PAGE: sodium dodecyl sulfate-polyacrylamide gel electrophoresis
SEC: size-exclusion chromatography
TEM: transmission electron microscopy
UPLC: ultra performance liquid chromatography.
